# Investigation of Functional Activity of Cells in Granulomatous Inflammatory Lesions from Mice with Latent Tuberculous Infection in the New *Ex Vivo* Model

**DOI:** 10.1155/2013/371249

**Published:** 2013-10-02

**Authors:** Elena Ufimtseva

**Affiliations:** The Institute of Biochemistry of the Siberian Branch of the Russian Academy of Medical Sciences, 2 Timakova Street, Novosibirsk 630117, Russia

## Abstract

The new *ex vivo* model system measuring functional input of individual granuloma cells to formation of granulomatous inflammatory lesions in mice with latent tuberculous infection has been developed and described in the current study. Monolayer cultures of cells that migrated from individual granulomas were established in the proposed culture settings for mouse spleen and lung granulomas induced by *in vivo* exposure to BCG vaccine. The cellular composition of individual granulomas was analyzed. The expression of the leukocyte surface markers such as phagocytic receptors CD11b, CD11c, CD14, and CD16/CD32 and the expression of the costimulatory molecules CD80, CD83, and CD86 were tested as well as the production of proinflammatory cytokines (IFN**γ** and IL-1**α**) and growth factors (GM-CSF and FGFb) for cells of individual granulomas. The colocalization of the phagocytic receptors and costimulatory molecules in the surface microdomains of granuloma cells (with and without acid-fast BCG-mycobacteria) has also been detected. It was found that some part of cytokine macrophage producers have carried acid-fast mycobacteria. Detected modulation in dynamics of production of pro-inflammatory cytokines, growth factors, and leukocyte surface markers by granuloma cells has indicated continued processes of activation and deactivation of granuloma inflammation cells during the latent tuberculous infection progress in mice.

## 1. Introduction

Tuberculosis, with the intracellular parasite *Mycobacterium tuberculosis* as the causative agent, accounts for about 2 million deaths annually and is one of the leading causes of deaths from infectious disease caused by a single agent. According to WHO reports, one-third of the world's human population is infected with *M. tuberculosis* and each infected with this bacterium has a 5–10% risk of developing active tuberculosis, which amounts to 8-9 million new cases annually. Intracellular presence of mycobacteria in specific inflammatory lesions called granulomas is one of the known features of the tuberculosis. Granulomas are the aggregates of the immune cells, predominantly macrophages, containing mycobacteria [1–4]. Granulomas on the one hand restrict dissemination of tuberculosis infection but, on the other hand, provide for latency with the chronic totally asymptomatic infection and set the stage for its reactivation [1, 5–7]. Macrophages are the cells of the innate immunity system. They are the components of the primary immune response acting to attack and kill infectious agents in phagolysosomes. However, sometimes the phagosomes with mycobacteria do not fuse with lysosomes in host cells, and so pathogens can still survive and replicate [6–11].

Cytokines play the most important role in controlling immune system function as a response to a contagion. It was shown that in tuberculosis infection, IFN*γ* is the most important reaction controller for both innate and adaptive immune responses [4, 5, 8, 12]. The IFN*γ*, produced mainly by T cells, supports inflammatory response progress, which also includes granuloma formation [5, 12]. The IFN*γ* activates macrophages leading to the increase in their phagocytal and microbicidal functions. It also promotes production of the proinflammatory cytokines (IL-1 and TNF*α*) and growth factors (GM-CSF and FGFb) that are involved in cell differentiation and aging in the inflammatory tissue. The IFN*γ* is also the most important transmitter in the elaboration of adaptive Th1-dependent response manifested by the increase of the expression of MHC class I and II proteins and costimulatory molecules CD80, CD83, and CD86 on the surface of antigen-presenting cells [12]. Macrophages, activated by IFN*γ*, absorb mycobacteria, opsonized by specific antibodies and complement, with the phagocytic receptors CD11b, CD11c, CD14, and CD16/CD32 and destroy them by means of producing antimicrobial effectors such as oxygen radicals and nitric oxide [4, 5, 7]. It was shown that the phagocytic receptors CD11b, CD11c, CD14, and CD16/CD32 on the surface of macrophages were activated by IFN*γ*. As observed in IFN*γ*-deficient mice, subjects with defects in IFN*γ* are profoundly susceptible to mycobacterial infections [5, 7]. Both forms of IL-1, predominantly cell-associated IL-1*α* and secreted IL-1*β*, are also considered to be basic transmitters of the inflammatory response. They have a wide range of biological potency, including the increase in immune cell resistance to bacterial infections via cytokine synthesis and growth-stimulating factor induction. GM-CSF, produced by activated macrophages, fibroblasts, and T-lymphocytes, is a hematopoietic factor that stimulates the development of neutrophils and macrophages and enhances their functional antibacterial activity, as well as takes part in proinflammatory cytokines synthesis induction [12, 13]. FGFb, a protein of the FGF family of growth factors, supports the migration of cells to inflammatory tissues and regeneration of tissues by promoting fibroblasts proliferation and differentiation that often leads to fibrotic lesions of granulomas [13].

During the last years the expression of surface markers and cytokines in bulk population of tuberculosis granuloma cells, obtained mostly from body organs of several mice and then treated with collagenase to destroy extracellular structures, was analyzed using flow cytometry method [14–23]. However, the characteristics of individual granulomas and their cells as well as the role they play in formation of granuloma inflammation within different stages of tuberculosis pathogenesis have not been studied much. The models of experimental tuberculosis infection in zebrafish (*Danio rerio*) juveniles and mice have been recently developed to analyze the individual granulomas [24–28]. Studies of transparent zebrafish juveniles infected with *M. marinum* allowed the analysis of the initial stages of the formation of macrophage granulomas and their responsiveness to antituberculous therapy [24–27]. Real-time imaging of individual granulomas in the livers of mice infected with the Bacillus Calmette-Guérin (BCG) vaccine prepared from a strain of attenuated live *M. bovis *allowed to describe the dynamic behavior of macrophages and lymphocytes especially their interaction within granulomas critical for the induction and regulation of antituberculous immunity [28]. However, there are many unanswered questions related to the emergence, development, and maintenance of granulomas made up by cells of different types and to the dissemination of the tuberculous infection to mammalian organs and tissues [7, 16]. Consequently, more research is needed for studying not only formation and growth but also mechanisms of the interaction between mycobacteria and host cells during latent tuberculous infection and after reactivation [6]. New systems and experimental models that are aimed to develop new antituberculous drugs intended for prevention and treatment of tuberculosis are required as well. 

In the present work the granuloma cells were studied using the new *ex vivo* culture system after the initial granulomas were induced in mice by infection with BCG vaccine *in vivo*. As a result, monolayer cultures of cells migrated from individual granulomas were established and maintained in the proposed culture settings. The dynamics of granuloma formation and functional contributions of granuloma cells of different types as well as with the analysis of surface markers, cytokines, and growth factors to promoting the growth of granuloma inflammation in mice with latent BCG infection has been studied using these granuloma cell cultures. 

## 2. Materials and Methods

### 2.1. Animals

BALB/c mice aged 2 months were obtained from the Animal Breeding Facility of the Institute of Cytology and Genetics, Siberian Branch (SB) of Russian Academy of Sciences (RAS), Novosibirsk, Russia, and kept under standard vivarium conditions in accordance with all the known guidelines for laboratory animal care. 

### 2.2. Chemicals and Antibodies

RPMI 1640, phosphate buffer saline (PBS), fetal bovine serum (FBS), glutamine, and gentamicin were obtained from BioloT (St. Petersburg, Russia). All other chemicals were obtained from Sigma-Aldrich (St. Louis, USA). APC-labeled antibody against mouse CD11b was obtained from eBioscience (San Diego, USA). All other primary specific antibodies, secondary specific antibodies labeled by biotin, and horseradish peroxidase conjugated streptavidin were purchased from BD Pharmingen (San Diego, USA). FITC-labeled antibodies against rat or mouse immunoglobulins (Ig) were obtained from Abcam (Cambridge, England). Tissue-Tek O.C.T. Compound was obtained from Sakura (the Netherlands). VECTASHIELD Mounting Medium with DAPI (4′,6-diamidino-2-phenylindole) was given as a gift by Vector Laboratories (Burlingame, USA). 

### 2.3. Infection of Mice

Mice were infected with the vaccine prepared from an attenuated live strain of *M. bovis *(the Bacillus Calmette-Guérin vaccine, BCG-1, Allergen, Stavropol, Russia) at a dose of 0.5 mg per mouse, which amounted to 3 × 10^6^ viable BCG-mycobacteria in 0.9% NaCl solution. Twenty-three mice were infected via tail vein injections with 100 *μ*L of the suspension per mouse and two mice were infected intraperitoneally with 200 *μ*L of the suspension per mouse. 

### 2.4. Histology

The spleens and lungs of mice were removed and cut into pieces in RPMI 1640 medium. Parts of tissues from some mice were collected and fixed with 4% formaldehyde solution in PBS (pH 7.4) for 90 min at room temperature. After fixation, the tissues were washed with PBS, incubated with 30% sucrose in PBS (pH 7.4) for 20 hours at +4°C, frozen in Tissue-Tek O.C.T. Compound at −25°C, and sectioned at 16 *μ*m slides on Microtome cryostat HM 505N (Microm, Germany) at the Shared Center for Microscopic Analysis of Biological Objects of the Institute of Cytology and Genetics, SB RAS, (Novosibirsk). Sections were air-dried on slides and stained with azure-eosin and methylene blue.

### 2.5. Isolation and Culture of Mouse Granulomas *Ex Vivo*

The isolation of granulomas from the spleens and lungs of mice of two groups after one and two months lasting infection was performed as described by Sacco and the colleagues [23] without the use of collagenase. In brief, the organs taken from animals or rests of tissues after prepared histological sections were cut in 5 mL of RPMI 1640 medium with 50 *μ*g/mL gentamicin into small pieces. For homogenization the dissected lungs were further disrupted by gently pushing the tissue through a metal screen with pores of 1 mm diameter. Granulomas were isolated from the organ homogenates by centrifugation at 150 ×g and washed three times in RPMI 1640 medium with 50 *μ*g/mL gentamicin. Granuloma pellets in a complete growth medium containing 10% FBS, 2 mM glutamine, and 50 *μ*g/mL gentamicin were placed at low-medium density to 24-well plates (Orange Scientific, Belgium) with cover glasses in the bottom and cultured in 0.5 mL medium for several days at +37°C in atmosphere containing 5% CO_2_. FBS inactivated by heating for 30 min at +56°C was used. 

### 2.6. Cell Staining

After 2–5 days of *ex vivo *culture, granuloma cells on cover glasses were fixed with 4% formaldehyde solution in PBS (pH 7.4) for 10 min at room temperature. Before staining, the preparations were kept in 0.4% formaldehyde solution in PBS (pH 7.4) at +4°C. To visualize acid-fast bacteria, some of the preparations with granuloma cells were washed with PBS and stained by Ziehl-Neelsen method according to standard protocols. The cells were further counterstained with 1% methylene blue. The other preparations were stained using antibodies raised against cell surface markers. The preparations of cultured and fixed, as granuloma cells, peritoneal macrophages of normal BALB/c mice without BCG infection were used as the control groups. The control fibroblasts were obtained by spontaneous cell migration from fragments of the lung tissue or the splenic capsule from intact BALB/c mice in the wells of 24-well plates with cover glasses on the bottom in minimal amount of a complete growth medium at +37°C in atmosphere containing 5% CO_2_. After removing tissue fragments the migrated fibroblasts on cover glasses were further cultured for optimal density in 0.5 mL medium for several weeks. Several markers were stained for using the preparations of peritoneal macrophages obtained from mouse 25 after two months of intraperitoneal BCG infection and cultured as granuloma cells. 

### 2.7. Antibody Staining

Cell preparations were washed with PBS, treated within 0.5–2 minutes in 0.3% Triton-X100 solution (only for cytokines staining), blocked in PBS solution containing 2% BSA, and finally incubated first with primary specific antibodies and then with secondary specific antibodies labeled by biotin or fluorochromes. The hamster primary antibodies to CD11c and IL-1*α* were diluted 1 : 10 and 1 : 50, respectively. The rat antibodies to IFN*γ*, GM-CSF, CD11b, CD14, and CD16/CD32 were diluted in 1 : 100, 1 : 25, 1 : 250, 1 : 50, and 1 : 50, respectively. The mouse primary antibodies to FGFb were diluted 1 : 100. The goat secondary polyclonal antibodies against rat Ig were diluted 1 : 25; the mouse secondary polyclonal antibodies against hamster Ig were diluted 1 : 50. Specific staining was visualized using horseradish peroxidase conjugated streptavidin in diaminobenzidine solution containing 0.05% H_2_O_2_. The cells were further counterstained with 1% methyl green. Fluorescent visualization of the proteins was enabled using FITC-labeled antibodies against rat or mouse Ig diluted 1 : 400. Some of the cell preparations were stained with antibodies against CD80, CD83, or CD86 labeled by PerCP-Cy5.5, APC, or PE-Cy7, respectively, diluted 1 : 100. Immunofluorescent staining of cells was analyzed using VECTASHIELD Mounting Medium with DAPI. After recording of the confocal images of the cells, the preparations were washed of VECTASHIELD Mounting Medium in PBS during 20 minutes and restained for acid-fast mycobacteria using Ziehl-Neelsen stain. 

### 2.8. Microscopy

The histological and cytological preparations were examined at the Shared Center for Microscopic Analysis of Biological Objects of the Institute of Cytology and Genetics, SB RAS, using an Axioscop 2 *plus* microscope (Zeiss) and objectives with various magnifications (Zeiss), photographed using an AxioCam HRc camera (Zeiss); the images were analyzed using the AxioVigion 4.7 microscopy software (Zeiss). Proteins stained with fluorescent dyes were examined under an LSM 510 or LSM 780 (Zeiss) confocal microscopes using the LSM Image Browser and ZEN 2010 software (Zeiss). 

### 2.9. Statistical Analysis

Statistical data processing was performed using MS Excel 2007 (Microsoft). Differences were tested for significance using Student's *t*-test.

## 3. Results

### 3.1. *Ex Vivo* Culture of Granulomas

**Figure 1:**
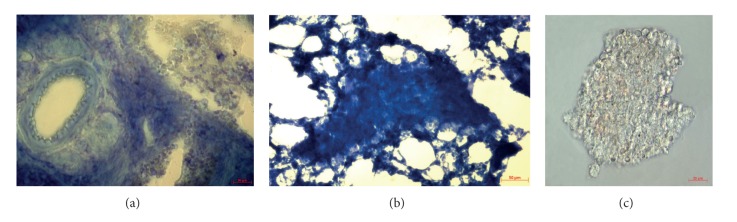
Solid granulomas (the central part of the image) in spleen (a) and lung (b) of BALB/c mice after one month of BCG infection were detected on histological sections with azure-eosin and methylene blue staining. (c) Phase contrast image of granuloma after isolation from mouse spleen. Scale bars: 20 *μ*m ((a), (c)), 50 *μ*m (b).

Granulomas were isolated from the spleens and lungs (S/and L/, resp.) of mice after one month (/1) and two months (/2) of being infected with the BCG vaccine and seeded into culture plates. Granulomas obtained at one month and two months after infection will be denoted as Gran/1 granulomas and Gran/2 granulomas, respectively, throughout. Gran/1 granulomas were from mice 1 ÷ 5; Gran/2 granulomas were from mice 1 ÷ 16 and 21 ÷ 25. Mice 1S/1 and 25S/2 were infected intraperitoneally; the others were infected via tail vein injections. By the time of isolation, none of the mice had been observed to have acute tuberculous disease. Therefore the granulomas were isolated from mice with latent chronic BCG infection. All granulomas examined in lungs and spleens of BCG-infected mice on histological sections were solid (Figures 1(a) and 1(b)). So as it is well-known, the solid granulomas are markers for tuberculous inflammatory lesions after experimental mycobacterial infection of BALB/c, C3H, or C57BL/6 mice [4, 5, 14–16, 20, 21, 23]. As it was noted, very small amount of granulomas was developed in mouse lungs after one and two months of BCG infection by intraperitoneal or via tail vein injections. 

**Figure 2:**
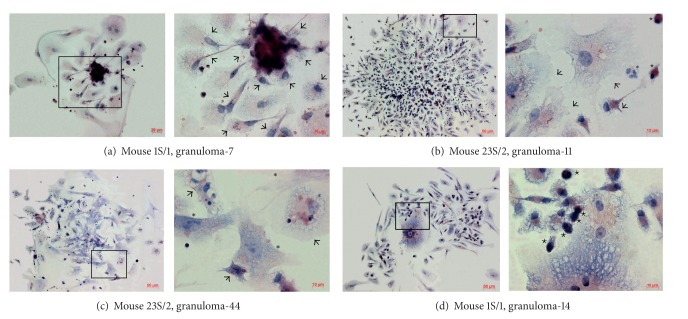
Granulomas from the spleens (S/) of mice that were infected with BCG vaccine* in vivo* for one and two months (/1 and/2, resp.) and after *ex vivo* culture for a few days. Ziehl-Neelsen staining for acid-fast mycobacteria. Scale bars: 50 *μ*m ((b)-(c), left panels, (d), left panel), 20 *μ*m ((a), left panel), 10 *μ*m ((a)–(c), right panels, (d), right panel). Granulomas with different cell types: pictures ((a)–(c), right panels, (d), right panel) are enlarged images of the areas defined by black boxes in pictures ((a)–(c), left panels, (d), left panel), respectively. ((a)–(c)) Macrophages with BCG-mycobacteria are indicated by black arrows. ((b), (d)) Dendritic cells are indicated by black stars. ((b)-(c)) A neutrophil and fibroblasts are indicated by black snowflakes, respectively. The other cells are macrophages without BCG mycobacteria, lymphocytes, and erythrocytes. (a) Macrophages that have migrated from a granuloma. (b)–(d) The monolayer cultures of cells migrated from individual granulomas. (b)-(c) Granulomas with an increased number of macrophages and fibroblasts, respectively. (d) Langhans giant cell in a granuloma. The fusion of macrophage membranes and entry of new macrophage nuclei to the nuclear aggregates of Langhans giant cell.

After isolation from mouse organs (Figure 1(c)), the granulomas were cultured *ex vivo* at low-medium density using heat-inactivated FBS without complement proteins for a varying number of days, from 2 to 5 on most occasions. Monolayer cultures of cells that had migrated from individual granulomas were established in the proposed culture settings (Figures 2(a)–2(d)). Cell migration from granulomas was first observed as early as on day 1. On day 2, monolayer cultures with fully distributed granuloma cells spread out on the slide were observed in the material from mice, although most granulomas still had aggregated granuloma cells in the center of monolayer cultures (Figure 2(a)). On days 3 and 4, most cells were observed to have migrated away from the granulomas (Figures 2(b)–2(d)). On day 5, most monolayer cultures had fully distributed granuloma cells. On week 3, the monolayer cultures of granuloma cells showed morphological signs of cell death. Granulomas from most mice were examined on days 2–4 after seeding for *ex vivo *culture. Any granuloma cell culture that appeared as a monolayer further will be called “a granuloma.” Granulomas were isolated from the spleens of all the BCG-infected mice. From the spleen of mouse 6S/2, diffuse leukocyte infiltrates and very few well-formed granuloma aggregates were obtained. Each mouse lung yielded a single granuloma or none at all. The number of granulomas obtained from the spleens varied significantly. In particular, while 1S/2, 3S/2 and 4S/2 mice yielded 411, 319, and 219 granulomas, respectively, mice 1S/1, 5S/1, 12S/2, and 13S/2 yielded as few as 48, 26, 58, and 33 granulomas, respectively. The average number of granulomas obtained from the spleens of the rest of the mice was in the range of 100 to 200. It was noted that the number of granulomas obtained from the spleens of BALB/c mice had correlated with its quantity (much or small) on histological sections of tissues. The acid-fast mycobacteria were detected in granuloma cells isolated from different organs of all examined mice in *ex vivo* culture (Figures 2(a)–2(d)). 

### 3.2. Granuloma Size in Mice with Latent Tuberculous Infection

Granuloma size in the spleens of mice of both groups, infected for one and for two months with BCG, was measured after seeding *ex vivo *culture before obtaining monolayers. It varied significantly: granulomas were ranging from 20 *μ*m to more than 300 *μ*m in length. The average granuloma length was in the range of 70 to 90 *μ*m. A significantly greater average length was in Gran/2 (90.89 ± 2.12, 1314 granulomas in 19 mice) than Gran/1 granulomas (76.22 ± 3.45, 113 granulomas in four mice) (*P* < 0.05). Interestingly, granulomas in zebrafish juveniles had an average length of 85–105 *μ*m on days 3–5 after being infected with *M. marinum *[21]. 

### 3.3. The Cellular Composition of Granulomas in Mice with Latent Tuberculous Infection

**Figure 3:**
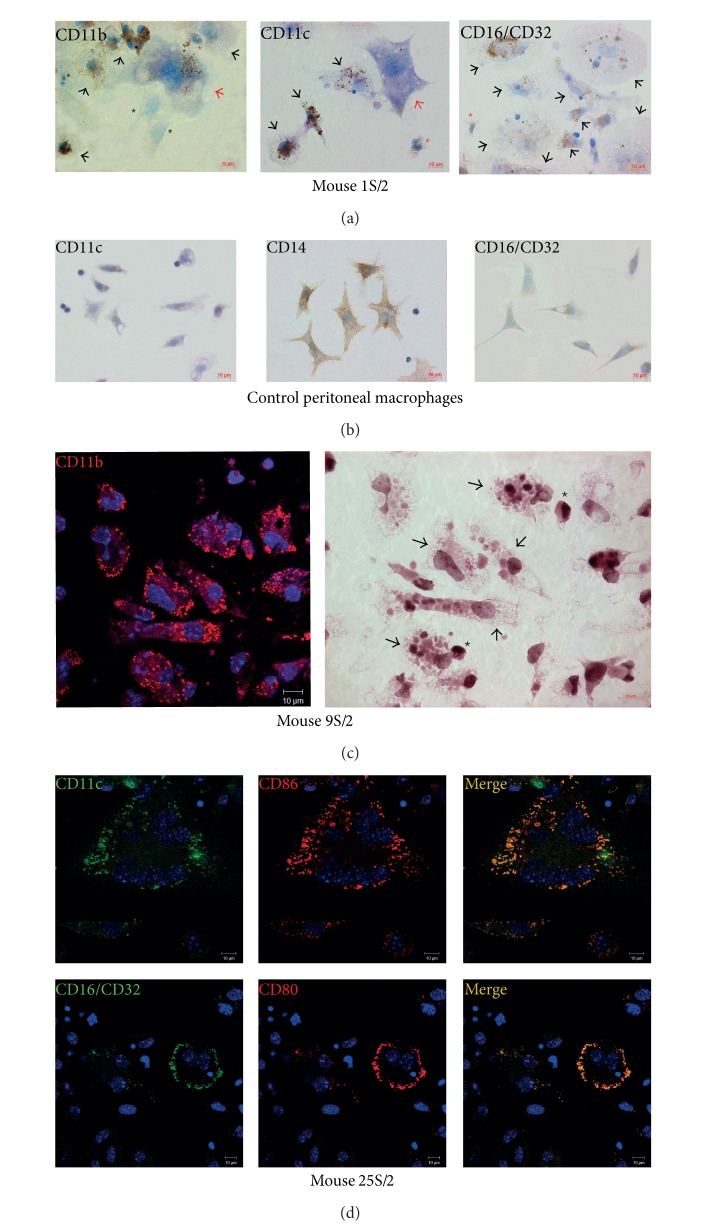
The phagocytic receptors and the costimulatory molecules on granuloma cells from the spleens (S/) of mice that were infected with the BCG vaccine *in vivo* for two months (/2) and after *ex vivo* culture for a few days. Scale bars: 10 *μ*m. Different parts of granulomas are shown. ((a)-(b)) The immunochemical localization of the leukocyte surface markers CD11b, CD11c, CD14, and CD16/CD32 on granuloma cells and control mouse peritoneal macrophages, respectively. The brown color of the cell membranes indicates the presence of the receptors in these regions. Black and red arrows indicate macrophages and megakaryocytes stained for cell surface markers, respectively; red stars indicate neutrophils specifically stained for the receptors; black stars indicate fibroblasts without the antigen. ((c)-(d)) The confocal immunofluorescent localization of the cell surface receptors CD11b (red signal), CD11c and CD16/CD32 (green signals), and CD80 and CD86 (red signals) in granuloma cells. Colocalization of the receptors on confocal images of cells (yellow signal). Staining nuclei with DAPI (blue signal). (c) On the right panel is the same granuloma part that is on the left panel, but it is restained to acid-fast BCG-mycobacteria by Ziehl-Neelsen method. Macrophages and dendritic cells with BCG-mycobacteria are indicated by black arrows and stars, respectively. ((d), top panels) A part of a granuloma with a Langhans giant cell.

The cellular composition was inferred for each granuloma individually. The granulomas were largely composed of macrophages varying in population size (Figures 2(a), 2(b), and 2(d)). All the macrophages had the lightly staining vacuolar cytoplasm with a large number of cell membrane protrusions (Figures 2(a)–2(d), 3(a), and 3(c)). Binucleated macrophages were a common occurrence. The macrophage population size in granulomas ranged from 15 cells to 200–400 cells. Basically, the average macrophage population size per granuloma ranged from 30 to 90 cells depending on the mouse. The difference between Gran/1 and Gran/2 granulomas in average macrophage population size did not reach statistical significance and was 60.01 ± 4.67 cells with 346 granulomas analyzed in five mice and 64.44 ± 2.96 cells with 857 granulomas analyzed in 20 mice, respectively. 

Besides macrophages, cells of nine more types were observed in the granulomas from mice with latent BCG infection: dendritic cells, lymphocytes, fibroblasts, neutrophils, eosinophils, megakaryocytes, thrombocytes, erythrocytes, and multinucleate Langhans giant cells. The population sizes of cells of other types were much lower and still varying therefore they were analyzed as the percentage out of the macrophage population in granulomas in Table 1. Because thrombocytes and erythrocytes were located on peripheral macrophages in spleen granulomas and were not found in lung granulomas, we did not consider counting these cells in the present work. While dendritic cells and lymphocytes were found in nearly all the granulomas examined, the cells of other types were observed in much fewer mouse granulomas (see Table 1). 

The few granulomas isolated from the lungs of different mice were found to have the same cellular composition as the granulomas isolated from the spleens. In the lung granulomas, macrophages were the major cell type, the dendritic cell population was the same as in the spleen granulomas, and fibroblasts showed an occasional occurrence. The main difference between mouse lung and spleen granulomas was that the former had extremely small lymphocyte populations.

The average dendritic cell population in the mouse spleen granulomas remained constant during latent BCG infection and was about 10% of the macrophage population whether in Gran/1 or Gran/2 granulomas (see Table 1). Dendritic cells were mostly smaller in size than macrophages. These cells had an increased number of tiny cell membrane protrusions, and their nuclei and cytoplasm were very densely stained by histochemical dyes (Figures 2(b) and 2(d)). All the dendritic cells were mononuclear.

A comparison made between Gran/1 and Gran/2 granulomas for the fibroblast populations revealed their significant, nearly twofold increase in Gran/2 (*P < 0.05, see Table 1). The number of fibroblasts on the periphery of Gran/2 granulomas was also increased as compared with that in Gran/1 granulomas (**P < 0.01). The percentage of fibroblasts in the examined granulomas did not exceed 15% of the macrophage populations, but granulomas with the fibroblast populations made up more than 40% of the observed macrophage populations (Figure 2(c)). 

The number of granulomas with polymorphonuclear leukocytes (neutrophils and eosinophils) had changed from mouse to mouse as well as the number of these cells within granulomas, the range being 5–13% of within-granuloma macrophage populations. Noteworthy, the neutrophils remained viable in all granuloma cell cultures (Figures 2(b) and 3(a)); even though when cultured in the medium without adding cytokines, they normally die within 24 hours [29]. The average number of neutrophils was almost equal in Gran/1 and Gran/2 granulomas, while eosinophils were largely found in Gran/1 granulomas (***P < 0.001, see Table 1).

Unexpectedly, we did observe multinucleate Langhans giant cells in some of the mouse granulomas, while they are known as the hallmarks of human granulomas [5, 30]. These cells were located peripherally or centrally in granulomas from the spleens of some mice with latent BCG infection, no matter whether delivered intraperitoneally or via tail vein injections. Cells with more than five nuclei were attributed to this cell type. The nuclei within these often giant cells could be arranged to form circles around the center of the cell or longitudinal strands (Figures 2(d) and 3(d)). Cells containing both 6–15 and up to 30–60 nuclei were observed. In *ex vivo* culture, these cells continued to form by fusion of the membranes of Langhans giant cells with adjacent macrophages (but not with dendritic cells) and by admission of new macrophage nuclei to the nuclear aggregates (Figure 2(d)). The difference between Gran/1 and Gran/2 granulomas from the mouse spleens in multinucleate Langhans giant cell population did not reach statistical significance (see Table 1).

In many of the mice infected with BCG via tail vein injections, 5–10% of spleen granulomas were identified as containing megakaryocytes (Figures 3(a) and 5(c)). These cells were often found being single on the periphery of granulomas. The increased number of granulomas, which contained megakaryocytes, was found in the leukocyte infiltrates from the mouse 6S/2 spleen. The megakaryocytes appeared as large, often roundish cells up to 60–90 *μ*m in length with the strongly stained cytoplasm, with or without long cytoplasmic protrusions, each with a giant oval or lobular polyploid nucleus (DNA content from 12 n to 64 n, a personal communication by T.D. Dubatolova). Occasionally, thrombocytes, formed due to cytoplasmic fragmentation, were observed in these cells (Figures 3(a) and 5(c)). The difference between Gran/1 and Gran/2 granulomas from the mouse spleens in the population of cells of this type did not reach statistical significance (see Table 1). A more detailed study of this cell type within granulomas with using antibodies against megakaryocyte antigens to characterize them at various stages of differentiation and functional state appears to be necessary.

Comparison of the cell composition of Gran/1 and Gran/2 granulomas from the mouse spleens has detected the increased amount of dendritic cells, neutrophils (by a decade), and megakaryocytes in leukocyte infiltrates, obtained from the spleen of the mouse 6S/2, while fibroblasts and eosinophils were not observed at all (see Table 1).

Our observation of the morphology of the macrophages, dendritic cells, segmented leukocytes, fibroblasts, megakaryocytes, and Langhans giant cells in the mouse granulomas cultured *ex vivo* suggests that none of those cells was apoptotic or necrotic. Few lymphocytes had segmented nuclei, while the integrity of the cell membranes was not compromised. 

### 3.4. Granuloma Cells with Leukocyte Surface Markers: Phagocytic Receptors and Costimulatory Molecules

Monocyte/macrophage-specific leukocyte surface markers (CD11b, CD14, and CD16/CD32) and leukocyte surface markers specific for dendritic cells (CD11c, CD80, CD83, and CD86) were found on both cells types within the granulomas examined (Figures 3(a), 3(c), and 3(d)). Examined markers were found on the cell surface of granulomas of both types with and without acid-fast BCG-mycobacteria (Figure 3(c)). The peritoneal macrophages from the control groups, obtained from intact mice or mouse 25 after two months of intraperitoneal infection with the BCG vaccine, did not stain for the integrin receptors CD11b and CD11c, that recognize the inactivated complement fragment C3b, the Fc receptor CD16/CD32, or the costimulatory molecules CD80, CD83, and CD86, while granuloma cells showed a strong microdomain staining for these antigens for all the examined preparations, and various receptors often colocalize (Figures 3(a)–3(d)). The maximum size of the microdomains was 1 *μ*m. CD14, the receptor for lipopolysaccharide in bacterial cell walls, occurred at an increased density on the membranes of the peritoneal macrophages in the control mice and macrophages and dendritic cells in the granulomas (Figure 3(b)). The cell surface markers analyzed were not detected on fibroblasts from the lungs or spleens in the control mice, nor were they found on granuloma fibroblasts. By contrast, all the polymorphonuclear leukocytes in the granulomas intensively expressed the phagocytic receptors CD11b, CD11c, CD14, and CD16/CD32 (Figure 3(a)). Few granuloma lymphocytes showed staining for the phagocytic markers; no costimulatory molecules CD80, CD83, or CD86 were detected on their membranes. Staining megakaryocytes for the phagocytic markers on some occasions revealed a small amount of microdomains on their surface (Figure 3(a)). Both phagocytic and costimulatory receptors occurred at an increased density on the surface of the Langhans giant cells (Figure 3(d)). Neither thrombocytes nor erythrocytes in the granulomas stained for any of the markers in question. 

**Figure 4:**
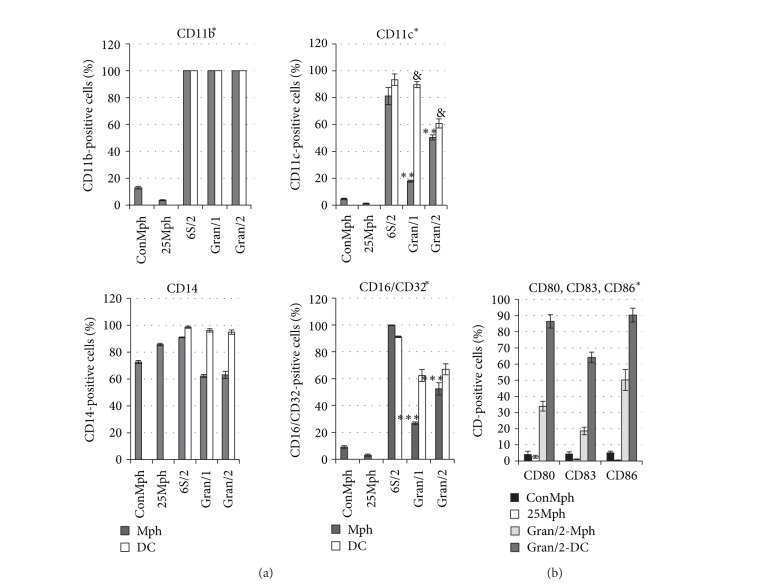
The macrophages (Mph) and dendritic cells (DC) with cell surface markers (a) CD11b, CD11c, CD14, CD16/CD32 and (b) CD80, CD83, CD86. The independently examined objects are control peritoneal macrophages (ConMph) from three intact mice, peritoneal macrophages obtained from mouse 25 (25Mph) after being intraperitoneally infected with the BCG vaccine for two months, cells in leukocyte infiltrates from mouse 6S/2 and granulomas from the spleens of three to five mice after one and two months (Gran/1 and Gran/2, resp.) of BCG infection *in vivo *and* ex vivo* culture after a few days. Gran/2 granulomas of 12 mice were analyzed for CD11b. Data are means ± SEM. *P < 0.001 (comparing CD-positive macrophages in controls (ConMph and 25Mph) and Gran/1 and Gran/2 granulomas), **P < 0.05, ***P < 0.01, ^&^ *P* < 0.01.

Granuloma cells (macrophages and dendritic cells) with expression of various surface antigens were analyzed in each granuloma separately, and the figures were averaged over the mice and then combined for Gran/1 and Gran/2 granulomas (Figures 4(a) and 4(b)). Many macrophages, observed in Gran/1 and Gran/2 granulomas from the spleens of all the examined mice, contained the phagocytic receptors CD11b, CD11c, CD16/CD32 and the costimulatory molecules CD80, CD83, and CD86, while the macrophages in the control groups did not (*P < 0.001). The average number of the macrophages that contained the phagocytic receptors CD11c and CD16/CD32 was largely found in Gran/2 granulomas, while dendritic cells stained for leukocyte surface marker CD11c were largely found in Gran/1 granulomas (Figure 4(a)). No difference between the control group and granuloma macrophages in the average population of CD14-positive cells was found. The granulomas had a higher percentage of dendritic cells stained for phagocytic markers CD11c, CD14, and CD16/CD32 than they had macrophage. No difference between Gran/1 and Gran/2 granulomas from the mouse spleens in the percentage of dendritic cells containing the phagocytic receptors CD11b, CD14, and CD16/CD32 was found (Figure 4(a)). All macrophages and dendritic cells of leukocyte infiltrates, obtained from the mouse spleen 6S/2, contained increased amount of the phagocytic receptors. On the whole, our study of leukocyte surface markers demonstrates significant activation of granuloma cells by the expression of the phagocytic receptors and costimulatory molecules both in leukocyte infiltrates, and in Gran/1 and Gran/2 granulomas of mice with latent BCG infection regardless of mycobacteria content in the cells. 

### 3.5. Analysis of Granuloma Cells for Production of IFN*γ*, IL-1*α*, GM-CSF, and FGFb

**Figure 5:**
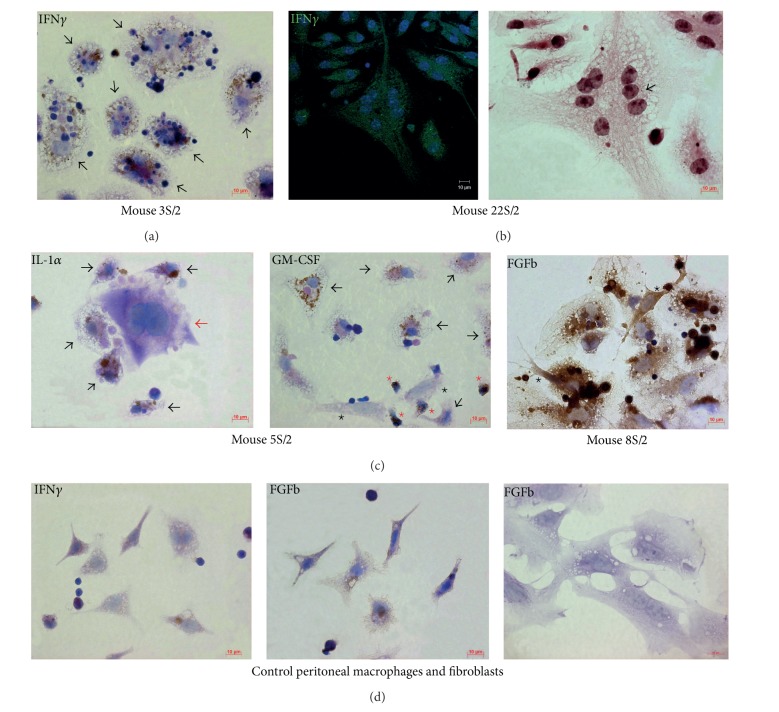
IFN*γ*, IL-1*α*, GM-CSF, and FGFb in granuloma cells from the spleens (S/) of mice that were infected with the BCG vaccine *in vivo* for two months (/2) and after *ex vivo* culture for a few days. Scale bars: 10 *μ*m. Detailed pictures of granulomas. ((a), (c)-(d)) Immunochemical localization of cytokines in granuloma cells ((a), (c)) and control mouse peritoneal macrophages ((d), left and central panels) and spleen fibroblasts ((d), right panel). The brown color of the cell membranes indicates the presence of cytokines in these regions. Black arrows indicate macrophages stained for (a) IFN*γ*, (c) IL-1*α*, and GM-CSF. (c) Red arrow indicates megakaryocyte; red stars indicate neutrophils with GM-CSF; black stars indicate fibroblasts without GM-CSF and with FGFb. (b) Confocal immunofluorescent localization of IFN*γ* (green signal) in granuloma cells. Staining nuclei with DAPI (blue signal). On the right panel is the same granuloma part as on the left panel but restained to acid-fast BCG-mycobacteria with Ziehl-Neelsen stain. BCG-mycobacterium in a Langhans giant cell is indicated by black arrow.

It is well established that cytokines IFN*γ*, IL-1, and GM-CSF are inflammatory mediators that activate and differentiate immune cells increasing their microbicidal potential in response to infection by intracellular pathogen thus playing an important role in initiating, regulating, and control of inflammation [3–5, 8, 12]. FGFb is involved both in increase and decrease in inflammatory response of organism by stimulating fibrotic granuloma changes. Immunocytochemical and immunofluorescence assay of IFN*γ*, cell-associated IL-1*α*, GM-CSF, and FGFb detected significant production activation for examined cytokines in granuloma macrophages and leukocyte infiltrates of mice after one and two months of being infected with BCG comparing to the peritoneal macrophages from the control group of untreated mice (*P < 0.001, Figures 5(a)–5(d)). Please note that macrophages and the Langhans giant cells with and without acid-fast BCG-mycobacteria were stained for IFN*γ* (Figure 5(b)), IL-1*α*, GM-CSF, and FGFb. The intensity of immunocytochemical staining for IFN*γ*, IL-1*α*, GM-CSF, and FGFb varied between cells (Figures 5(a) and 5(c)); therefore, different degrees of cytokine production by granuloma macrophages were probably demonstrated. Examined markers were confirmed in cytoplasm of neutrophils as well (Figure 5(c)). Cytoplasm of only small number of dendritic cells was stained for IFN*γ* in the present study. Production of IL-1*α* and GM-CSF in granuloma dendritic cells, lymphocytes, fibroblasts, and megakaryocytes was not demonstrated at all (Figure 5(c)). It was also shown that granuloma fibroblasts did not produce IFN*γ*. Almost all of the macrophages, dendritic cells, and fibroblasts in Gran/1 and Gran/2 granulomas were intensively stained for FGFb, whereas production of it in control cell cultures was not detected (Figures 5(c) and 5(d)). 

**Figure 6:**
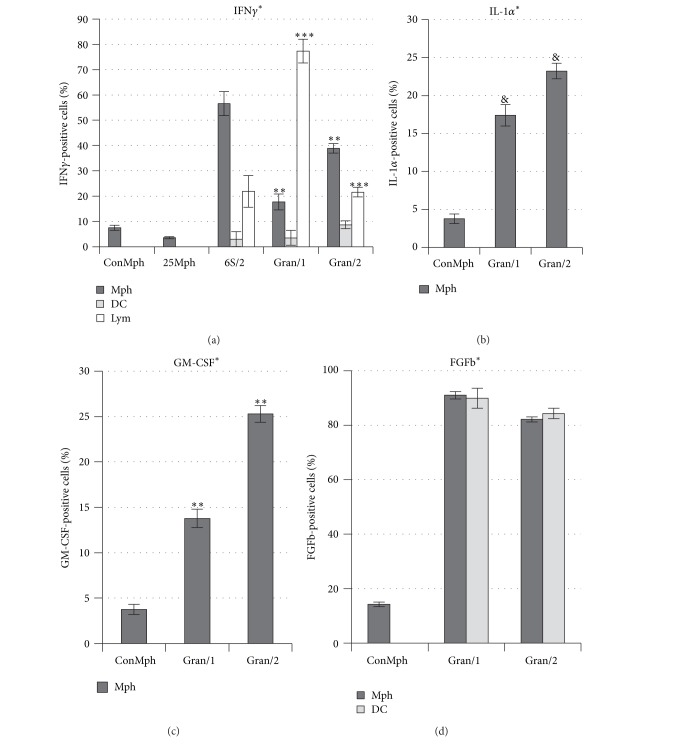
The macrophages (Mph), dendritic cells (DC), and lymphocytes (Lym) with production of (a) IFN*γ*, (b) IL-1*α*, (c) GM-CSF and (d) FGFb. The independently examined objects are control peritoneal macrophages (ConMph) from three intact mice, peritoneal macrophages obtained from mouse 25 (25Mph), that was intraperitoneally infected with the BCG vaccine for two months, cells in leukocyte infiltrates from mouse 6S/2 and granulomas from the spleens of three to six mice after one and two months (Gran/1 and Gran/2, resp.) of BCG infection *in vivo* and *ex vivo* culture after a few days. Data are means ± SEM. *P < 0.001 (comparing cytokine-positive macrophages in controls (ConMph and 25Mph) and Gran/1 and Gran/2 granulomas), **P < 0.01, ***P < 0.001, ^&^ *P* < 0.05.

Granuloma cells producing cytokines were analyzed for Gran/1 and Gran/2 granulomas in Figure 6. Significant variation in the amount of IFN*γ*-producing lymphocytes among the examined groups of cells was observed. The amount of lymphocytes synthesizing IFN*γ* in infiltrates of the mouse 6S/2 and Gran/2 granulomas was 20% on average, while 80% of lymphocytes in Gran/1 granulomas were stained for IFN*γ* (***P < 0.001, Figure 6(a)). The production of IFN*γ* in macrophages of Gran/1 and Gran/2 granulomas showed inverse dynamics: while almost 40% of cells in Gran/2 granulomas were producing IFN*γ*, only 20% of such macrophages were detected in Gran/1 granulomas (**P < 0.01, Figure 6(a)). The number of macrophages producing IL-1*α* and GM-CSF was increased in Gran/2 granulomas as compared with Gran/1 granulomas (^&^ *P* < 0.05 and **P < 0.01, resp.), though their amount in Gran/2 granulomas did not exceed 25% at the average (Figures 6(b) and 6(c)). The modulation in production of proinflammatory cytokines IFN*γ* and IL-1*α* and growth factor GM-CSF by macrophages and lymphocytes in Gran/1 and Gran/2 granulomas of mice with latent BCG infection was found as the result of performed analysis. Noticeably, the cytokine-producing macrophages also contained acid-fast BCG-mycobacteria in cells. 

## 4. Discussion

It is well known that the macrophages are the main modulators of tuberculosis infection in animals and humans affected by mycobacteria [4]. On the one hand, they contain the infection within granulomas thus preventing dissemination, but on the other hand, they keep latent chronic infection with a risk of reactivation as an acute tuberculous process [6]. It is important to remember that granulomas are complex inflammations containing not only macrophages, but cells of other types as well that can affect latent tuberculous infection progress in human or animal organism [3–8, 31].

The approach that we used for culturing mouse granulomas *ex vivo* allowed us to determine the input of cells of other types to the progress of granulomatous inflammation in mice with latent BCG infection. Granulomas isolated from the spleens of BALB/c mice after one and two months of being infected with BCG vaccine were markedly heterogeneous for their number and type (compacted or loose aggregates or diffuse leukocyte infiltrates), size, and the number of cells in them both within each mouse and between the mice. Interestingly, diffuse leukocyte infiltrates containing increased numbers of dendritic cells and neutrophils, which are the hallmarks of acute rather than chronic tuberculous infection, were observed even in the spleen of mouse 6S/2 infected with BCG for two months, where the number of well-formed granulomas was the lowest. It is possible that either the initial stages of granuloma formation in the mouse spleen or, on the contrary, developing active tuberculosis after short-term chronic tuberculosis were observed. Every hypothesis leads to additional investigations. As it is well known, most of BALB/c mice are resistant to developing active tuberculosis [32], but both individual properties of the host organism and its specific interactions with the particular microorganism may have an influence on the development of the primary immune response to BCG mycobacterial infection and its pathogenesis.

The question as to whether the granuloma structure remains closed at all times during latent infection or otherwise is one of the most critical issues in the matter of understanding mycobacterial dissemination to organs and tissues. It has recently been demonstrated that macrophages were highly mobile at the initial stage of tuberculous infection in zebrafish juveniles [25, 33], while in latent BCG infection these cells occurred in mouse hepatic granulomas as immobile scaffolds with only lymphocytes allowed to move around within [28]. Nevertheless, we have found in our studies that all the granuloma cells that were characteristic of latent BCG infection could successfully migrate from the granulomas under certain *ex vivo* culturing conditions involving heat-inactivated FBS without complement proteins. It can therefore be hypothesized that, under certain conditions within the organism, these cells can also actively travel across the organs and tissues and disseminate mycobacteria to them. It is possible that the difference in the number of granulomas isolated from the spleens of different mice is indicative of the different abilities of cells in migrating from granulomas in some mice and, consequently, in forming new granulomas within the same organ. It is also possible that the different sizes of these aggregates could be due to the different times of granuloma formation: larger granulomas must be older, while the smaller formed later from cells that had migrated from the older. However, these possibilities can still only be hypothesized upon due the insufficient knowledge of the processes underlying the emergence, development, optimal size, cellular composition of granulomas, and cell rotation, which are required, on the one hand, for restraint of infection and survival of the host and, on the other hand, for the replication and dissemination of the pathogen—such that the delicate trade-off is reached in the latent tuberculous infection. 

Our studies confirm that macrophages did play a key role in the pathogenesis of latent tuberculous infection in the mouse spleen and lung [4, 5, 8, 10–12, 31]. Dendritic cells, too, have necessarily been involved in the development of BCG-induced chronic inflammation in various organs of mice [7]. Eight more cell types have been identified in granulomas: fibroblasts, neutrophils and eosinophils, lymphocytes varying in size, erythrocytes, Langhans giant cells formed by macrophages, megakaryocytes and thrombocytes. All of them were parts of the granulomas and were largely confined to the periphery. It should be noted that neither histological studies nor *in vitro* models have ever revealed megakaryocytes in granulomas [4, 14, 15, 21]. It is suggested that multinucleate Langhans giant cells are very seldom observed in mouse granulomas [5, 30], while it is opposite for human granulomas [2–5, 12]. However, we did observe multinucleate Langhans giant cells containing up to 60 nuclei in some of the mouse granulomas. Generally we did not find differences in cellular composition between Gran/1 and Gran/2 granulomas, which suggest that, although its components varied from one granuloma to the next, their cellular composition was on the whole quite stable and had probably been established shortly after the mice had latent BCG infection. An increase in the fibroblast population in the Gran/2 BCG-induced mouse granulomas may have signaled the onset of gradual fibrosis of these structures. The contribution of the above-mentioned cells to the protection of the host from BCG infection remains to be known, and this knowledge is wanted as it will lead to a better understanding of the mechanisms underlying the persistence of granulomas in animal organs and tissues. 

The fact that dendritic cells were present in all the mouse granulomas suggests that this cell type is important for the development of latent BCG infection. The dendritic cell population is estimated as not higher than 0.1% of blood mononuclear cells in animals [34]. However, dendritic cells in the mouse granulomas made up from 5% to 25% of the granuloma macrophage population. Therefore, our studies confirm that dendritic cells are for some reason absolutely required for granulomas [7, 35, 36]. It is presumed that dendritic cells, as some of the main antigen-presenting immune cells, can both stimulate and suppress the immune response during tuberculous infection [7]. Dendritic cells are considered the principal disseminators of infection to animal organs and tissues, which results in miliary tuberculosis [7, 35, 36]. However, the factors accounting for the role of the dendritic cells in the granulomas and whether this role is subject to change during latent tuberculous infection have yet to be known.

Phagocytic and costimulatory markers were found on the membranes of macrophages and dendritic cells in the mouse granulomas but not on the control peritoneal macrophages (with the exception of the receptor CD14). The active status of cells in the mouse granulomas was indicated by both higher expression of the leukocyte surface markers and a microdomain organization of these receptors on the granuloma cells and their colocalization with each other in most microdomains on granuloma cell membranes. The question as to whether the observed microdomains are rafts and, therefore, places for transducing cellular signals to the effectors below, which modulate the behavior of mouse granuloma cells in latent BCG infection, has yet to be answered. As is known, to be able to ingest bacteria, the host's cells should have phagocytic receptors clustered on their membranes [13]. Therefore, the microdomain organization of the phagocytic receptors CD11b, CD11c, CD14, and CD16/CD32 on the surface of mouse granuloma cells suggested that the macrophages and dendritic cells were probably in a position to ingest mycobacteria in the granulomas and then have various signal transduction pathways activated [7, 8, 13, 35–37]. Detected differences in the amount of granuloma macrophages with CD11c and CD16/CD32 and dendritic cells with CD11c in Gran/1 and Gran/2 granulomas are held to imply expression modulation of these markers during the process initiation and growth of granulomatous inflammation in mice after being infected with BCG vaccine, though these findings demand for more investigations. In accordance with our findings recent data by Schreiber et al. [38] showed that in mouse granulomas induced by BCG-mycobacteria amount of dendritic cells with expression of different phagocytic and costimulatory molecules changed throughout chronic tuberculous infection. The increased amount of costimulatory molecules CD80, CD83 and CD86, which we detected on macrophages and dendritic granuloma cells, indicates their close involvement in initiating T-lymphocyte activation, that, as is known [3–5, 8, 28], is needed for the induction and regulation of antituberculous immunity. As leukocyte surface markers were detected on macrophages and dendritic granuloma cells, both with and without BCG-mycobacteria, it is necessary to proceed further analysis of the intracellular pathogen impact to the expression of the phagocytic receptors and costimulatory molecules by the cells of granulomatous inflammation in mice with latent tuberculous infection.

Studying of cytokine production in granuloma cells showed modulation of expression of IFN*γ*, IL-1*α*, and GM-CSF in macrophages during the progress of  latent tuberculous infection in mice. If more than a half of leukocyte infiltrate macrophages of the mouse 6S/2 were producing IFN*γ*, then there were less such cells detected in Gran/1 granulomas, but the amount of macrophages producing IFN*γ* significantly increased in Gran/2 granulomas. Synthesis of IL-1*α* and GM-CSF was detected mostly in macrophages of Gran/2 granulomas, and it shows that there was also ongoing activation of macrophages during the progress of the granulomatous inflammation in mice with latent tuberculous infection. It is probably the granuloma cells producing GM-CSF and FGFb growth factors that promoted their survival in both compact structures of granulomas and *ex vivo* culture. Inverse dynamics of producing IFN*γ* was studied in granuloma lymphocytes: almost all of the lymphocytes of Gran/1 granulomas contained IFN*γ* in cytoplasm; however, there were very few lymphocytes producing IFN*γ* detected in Gran/2 granulomas. IFN*γ*, released by T lymphocytes, is considered to activate macrophages and dendritic cells in human and animal granulomas for struggling with tuberculous infection [4, 5, 7, 8, 12]. Thus there were different granuloma cells having the amount of IFN*γ* production changed, that were observed during the progress of latent tuberculous infection in mice. However the reason of these changes is yet to be studied. Generally the investigation of synthesis of cytokines and growth factors in the granuloma cells, with or without BCG-mycobacteria, during the granulomatous inflammation in mice is necessary. 

 It is worth noting that the production of IFN*γ* and other cytokines, phagocytic and costimulatory markers in granuloma macrophages and dendritic cells, with or without acid-fast BCG-mycobacteria, were detected. Therefore, some of the activated macrophages and maturated dendritic cells of mouse granulomas did not manage to destroy intracellular BCG-mycobacteria. This observation disagrees on the issue that activated macrophages and maturated inflammatory dendritic cells must successfully destroy intracellular bacilli [3–5, 7, 12, 35, 36]. Basically the impact of granuloma cells infected by intracellular mycobacteria to the process of synthesis of proinflammatory cytokines, growth factors, the phagocytic receptors, and costimulatory molecules, is needed to be studied. It is extremely important to find out and analyze the reasons by which cells that are activated by many markers are still unable to destroy intracellular pathogen. 

## 5. Conclusions 

With the *ex vivo* model system developed we studied the impact of individual granuloma cells to growth and control of the granulomatous inflammatory lesions in mice with latenttuberculous infection. Granulomas from the mouse spleens and lungs were largely composed of macrophages; however, they also contained dendritic cells, lymphocytes, and occasionally fibroblasts, neutrophils, eosinophils, megakaryocytes, and multinucleate Langhans giant cells formed by fused macrophages. It has been demonstrated that all polymorphonuclear leukocytes, many macrophages, dendritic cells, lymphocytes, megakaryocytes, and Langhans giant cells of mouse granulomas, with or without BCG-mycobacteria, substantially expressed the phagocytic receptors, costimulatory molecules, and proinflammatory cytokines on high level. Also the modulation of the activation of granuloma cells (macrophages, dendritic cells, and lymphocytes) during the progress of the granulomatous inflammation in mice with latent tuberculous infection was detected. Therefore the suggested new *ex vivo* model of granuloma cell cultures allowed not only to evaluate the input of cells of different type to the growth of inflammatory granulomas by its functional means but also to start studying the intercourse of mycobacteria, the intracellular pathogen, and host cells during latent stage of tuberculosis infection in mice. The reported study helps to elucidate why vaccines and therapeutic treatment seldom rid the organism of tuberculous infection.

## Figures and Tables

**Table 1:** The cellular composition of granulomas from spleens of mice after one (Gran/1) and two months (Gran/2) of being infected with BCG vaccine.

Granuloma cells	No. of granulomas with cells		No. of cells in		
	Gran/1	Gran/2	Leukocyte infiltrations from mouse 6S/2	Gran/1	Gran/2
Macrophages	100.0	100.0	100.0	100.0	100.0
Dendritic cells	94.83 ± 1.43	94.12 ± 1.04	18.62 ± 2.64	9.11 ± 0.44	10.41 ± 0.3
Lymphocytes	93.32 ± 1.27	86.61 ± 3.69	40.24 ± 4.89	33.15 ± 2.71	34.49 ± 1.64
Fibroblasts	6.87 ± 4.0*	19.59 ± 3.52*	—	5.64 ± 1.17**	9.95 ± 0.65**
Neutrophils	10.51 ± 5.13	27.1 ± 7.64	29.2 ± 4.88	1.85 ± 0.09	2.59 ± 0.15
Eosinophils	26.84 ± 6.89	14.27 ± 3.26	—	7.5 ± 0.87***	2.33 ± 0.17***
Megakaryocytes	11.56 ± 7.23	7.81 ± 3.04	7.33 ± 0.36	2.03 ± 0.73	1.48 ± 0.13
Langhans giant cells	3.1 ± 2.62	2.32 ± 1.52	—	1.5 ± 0.12	0.89 ± 0.04

Data is presented as mean percentage of the number of granulomas containing a particular cell type out of the total number of examined granulomas or a particular cell type, out of the macrophage population in the granulomas and the standard error of mean of Gran/1 (5 mice, 346 granulomas) and Gran/2 (20 mice, 857 granulomas). *P < 0.05, **P < 0.01, ***P < 0.001.
